# Glued to Which Face? Attentional Priority Effect of Female Babyface and Male Mature Face

**DOI:** 10.3389/fpsyg.2018.00286

**Published:** 2018-03-06

**Authors:** Wenwen Zheng, Ting Luo, Chuan-Peng Hu, Kaiping Peng

**Affiliations:** Department of Psychology, Tsinghua University, Beijing, China

**Keywords:** babyface, visual selective attention, facial configuration, facial gender, consciousness

## Abstract

A more babyfaced individual is perceived as more child-like and this impression from babyface, as known as babyface effect, has an impact on social life among various age groups. In this study, the influence of babyfaces on visual selective attention was tested by cognitive task, demonstrating that the female babyface and male mature face would draw participants’ attention so that they take their eyes off more slowly. In Experiment 1, a detection task was applied to test the influence of babyfaces on visual selective attention. In this experiment, a babyface and a mature face with the same gender were presented simultaneously with a letter on one of them. The reaction time was shorter when the target letter was overlaid with a female babyface or male mature face, suggesting an attention capture effect. To explore how this competition influenced by attentional resources, we conducted Experiment 2 with a spatial cueing paradigm and controlled the attentional resources by cueing validity and inter-stimulus interval. In this task, the female babyface and male mature face prolonged responses to the spatially separated targets under the condition of an invalid and long interval pre-cue. This observation replicated the result of Experiment 1. This indicates that the female babyface and male mature face glued visual selective attention once attentional resources were directed to them. To further investigate the subliminal influence from a babyface, we used continuous flash suppression paradigm in Experiment 3. The results, again, showed the advantage of the female babyfaces and male mature faces: they broke the suppression faster than other faces. Our results provide primary evidence that the female babyfaces and male mature faces can reliably glue the visual selective attention, both supra- and sub-liminally.

## Introduction

The babyface usually refers to adult faces that have a facial feature similar to that of infants (Berry and McArthur, 1985). It is usually defined as a round face with big eyes, high raised eyebrows, a narrow chin and a small nose. All these features tend to evoke stereotypes, in the form of child-like traits, such as being naïve, cute, and warm, etc. (Berry and McArthur, 1985; McArthur and Berry, 1987; Zebrowitz and Montepare, 1992, 2005; Zebrowitz et al., 1992, 1993, 2007, 2012, 2015; Albright et al., 1997; Zebrowitz, 2006). The impression from babyface has an impact on various age groups (Zebrowitz and Franklin, 2014; Zebrowitz et al., 2015) and several aspects of social life (Zebrowitz and McDonald, 1991; Zebrowitz et al., 1992, 1998; Collins and Zebrowitz, 1995; Zebrowitz and Montepare, 2008; Livingston and Pearce, 2009; Poutvaara et al., 2009), which is known as babyface effect. For example, in a congressional election, a babyfaced candidate may lose to his more mature-faced looking opponent (Zebrowitz and Montepare, 2005). In small claims court, babyfaced litigants were more likely to get “benefit” and “protected” (Zebrowitz and McDonald, 1991; Zebrowitz and Montepare, 2008). Cross-cultural studies have identified similarities in babyface phenomena in different cultural contexts (Zebrowitz et al., 1992, 1993, 2012), but cultural and gender biases have been proposed, suggesting that the definition of the babyface in terms of facial structures and social perceptions varies across cultures (Zheng et al., 2016).

We conducted a study to research the babyface effect of Chinese faces and found that the definition and impressions of the Chinese babyface revealed cultural differences and gender biases. Chinese babyfaces have a lower forehead and closer pupil distance and look healthier. Chinese babyface tended to be perceived as more babyfaced for American participants, but more competent for Chinese participants. When evaluating the babyfacedness of a face, Chinese are more concerned about the combination of all facial features, whereas American are more sensitive to some highlighted babyfaced features (Zheng et al., 2016). Besides, for Chinese participants, facial gender affects the social perceptions of babyface. Zheng et al. (2016) have found that for male Chinese faces, both Chinese and Americans believe that the babyface shows less competence than mature faces. But for the female Chinese faces, Chinese consider the female babyface as more competent, but it is judged to be less competent by American subjects.

Though the babyface effect on our social life has been well studied, the attentional process of a babyface is still unknown to us. It is investigated that faces have an advantage in retaining attention (Bindemann et al., 2005). For this reason, we suspect that babyface, faces with special structure, highly possibly capture attention. Zebrowitz et al. (2009) suggested that the babyface effect comes from human’s preferences for babies. The appearance of the baby is called Kindchenschema (baby schema) (Lorenz, 1943), and it can induce positive emotions and help establish attachment, which is similar to what happens in the babyface effect (Dou et al., 2014). fMRI results have shown that the amygdala and fusiform face area (FFA) are the brain areas related to the babyface. When participants observe adults’ faces, their amygdala and FFA are more active with babyface than mature faces (Luevano and Zebrowitz, 2007; Zebrowitz et al., 2009). Similar results were found on baby’s faces (Bechara et al., 2000; Leibenluft et al., 2004; Kringelbach et al., 2008). The attention capturing effect is discovered on baby schema (McCall and Kennedy, 1980). In a variant of the dot probe paradigm, it is found that infant faces can be rapidly and perhaps automatically processed (Brosch et al., 2007), but the effect is limited to own-race infants (Hodsoll et al., 2010). However, we cannot get answers to what we really concern. The most frequently used methods on the babyface, such as self-report questionnaires and scales, have limitations in preventing participants from guessing experimental objectives, the expectation effect, and other confounding variables. It is lack of evidence, especially proof from cognitive behavior experiments, about whether an adult’s babyface will glue your eyes, supra- and sub- liminally drawing on one’s attention so that you can’t take your eyes off.

It is widely known that a babyface contains a certain configuration of facial features (e.g., a round face, high raised eyebrows, a narrow chin, and a small nose). The facial configuration is processed at the early stage of visual processing (Wang et al., 2017); the influence of a babyface may occur when visual selective attention is available. The processing of facial information is specific. Faces are detected and categorized faster than many other stimuli. Facial information can be processed more rapidly than other information (Palermo and Rhodes, 2007). What’s more, participants are able to encode some facial information without awareness (Pessoa et al., 2005). However, the facial processing may not be automatic; it probably requires specific attentional resources (Ricciardelli et al., 2016; Yan et al., 2017). Palermo and Rhodes (2002, 2007) reveal that the attentional resources are necessary on holistic face perception. Therefore, attentional resources may be needed to detect the low-level feature of a face, such as the facial configuration.

According to these findings, we can confidently assume that at the supra- and sub-liminal level, visual selective attention involves during the processing of the babyface and that visual selective attention is essential to achieving the babyface effect. Thus, we propose that the babyface influences our behavior by affecting visual selective attention. We conducted three cognitive behavior experiments to research the relationship between the babyface and visual selective attention on both supra- and sub- liminal level using facial gender as an independent variable. Reaction time and accuracy are more objective than self-report questionnaires and scales in helping us study the babyface. In Experiment 1, with a simple detection task, we attempted to detect if the babyface will attract the attention without intervening in participants’ visual selective attention. In Experiment 2, we made use of an experimental paradigm created by Sui and Liu (2009) to research whether a babyface presented outside foveal vision can capture attention in a spatial cuing task. We proposed that the babyface spontaneously competes with an ongoing cognitive task for spatial attention. And, it is also worthy to study whether the babyface has an advantage in breaking suppression. In Experiment 3, Continuous flash suppression (CFS) was referred because it is more effective than traditional rivalry suppression. We anticipate that the ability to attract attention with the babyface should also work without consciousness. The babyface should break suppression faster than mature faces.

## Experiment 1

### Materials and Methods

#### Participants

Forty-four undergraduate students from Tsinghua University, (28 females; ages 18–31 years; *M* ±*SD*, 23.20 ± 3.44 years) participated in the experiment. All participants had normal or corrected normal vision and were paid for their participation. The study was approved by the Tsinghua University Institutional Review Board and all participants gave informed consent.

We followed previous studies to decide our sample size. When we look back, the sample size is enough because we tested it using G^∗^Power 3.1.9.3 (Faul et al., 2007, 2009). To get a reasonable estimation of the effect size, we referred to a recent meta-analysis on attentional bias for positive as compared with neutral stimuli (Pool et al., 2016), which showed that the effect is Hedges’ *g* = 0.258. With the help of Lenhard and Lenhard (2016), we transformed it into *f* = 0.129. Using this effect size, we did the power analysis (*α* = 0.05, power = 0.80) and found that at least 43 participants were needed. The following experiments followed the same rule.

#### Material

In this study, Chinese faces were used as experiment material after being filtered, measured, rated and edited by Photoshop. These photos came from the Chinese Academy of Sciences (CAS) – Pose, Expression, Accessories, and Lighting (PEAL) Large-Scale Chinese Face Database, including 1040 adult volunteers (445 women) (Gao et al., 2008). The black-white photo group with unified background, light, focal length, neutral expression and no ornaments was chosen. The chosen faces are between the age of 22 and 45 years old.

A website^1^, utilizing machine learning techniques and the results of Zheng et al. (2016), was designed to measure the babyfacedness of Chinese faces. 147 female faces (72 babyfaces, 75 mature faces) and 170 male faces (82 babyfaces, 88 mature faces) with similar perceived age were selected as stimuli by this website and human evaluation^2^. The hair of these faces was removed and edited into 201 pixels × 252 pixels by Photoshop on a 17 inch LCD monitor (1024 × 768, 75 HZ), which has a gray background color (RGB: 128, 128, 128). It is reliable that the attractiveness of faces between two groups (babyface and mature face) of all genders has no significant difference, the babyfacedness between two groups with the same gender is significantly different and no significant difference between female and male faces with the same babyfacedness level^3^.

#### Stimuli

**FIGURE 1:**
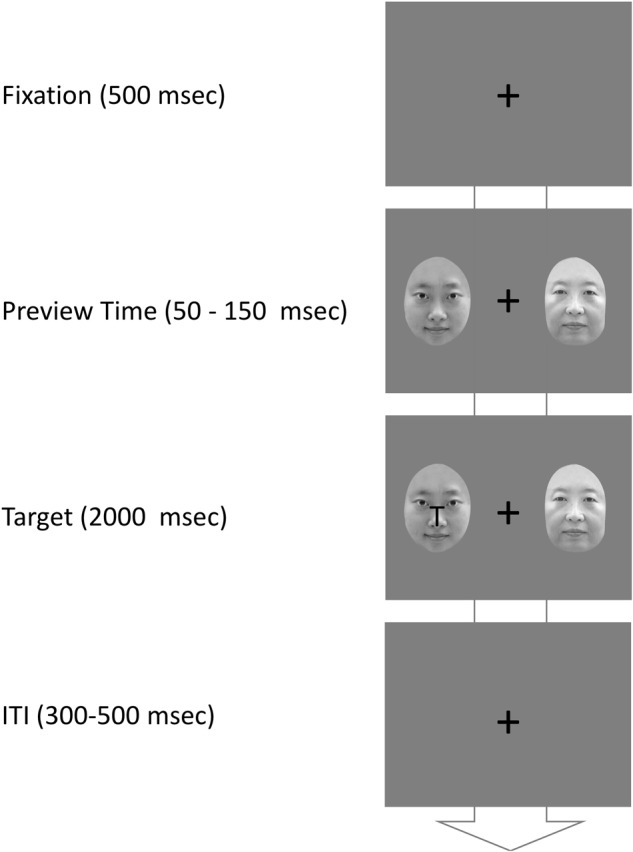
Illustration of the procedure used in the Experiment 1.

A central fixation point and two 3.8° × 4.5° faces were presented (see **Figure 1**). The distance between the center of the display and the outer edge of each face measured 5.5° of visual angle. A target letter “T” (0.7° × 0.7°) was presented in the center of a face (see **Figure 1**). The letter “T” was shown upright either on the left or right. E-Prime 2.0 (Psychology Software Tools, Inc.) was used to control the flow of the experiment and to collect response data. Participants were tested individually.

#### Procedure

The participant’s viewing position was set about 65 cm away from the computer monitor. The experimental procedure is illustrated in **Figure 1**. Each trial began with a central fixation cross for 500 ms. Two faces (a babyface and a mature face) with the same gender were randomly selected and they were presented in the bilateral visual fields for a randomized preview time between 50 and 150 ms. Following the preview display, a target letter overlapped in the center of one face. The participants were asked to press the “Space Bar” when the target “T” was presented. This display was presented until a response or 2000 ms. The inter-trial interval (ITI) was randomly set between 300 and 500 ms. Participants had to pass the practice experiment with an accuracy over 90% before they took the 320 experimental trials. The target was presented in 80% trials. The remaining 20% trials did not display target, which were catch trials. Each of the four conditions (2 Face gender × 2 Target match: Target on babyface, Target on mature face) in the experimental trials had 64 trials. Participants were given short breaks after every 40 trials. It took about 20 min to finish the experiment.

### Results

No participant was excluded since their mean reaction time and accuracy all fall within three standard deviations of the sample. In the analysis of mean reaction times, trials with correct responses as well as reaction times in three standard deviations in each condition for individual participant were included. We conducted a Repeated Measures of 2 (Face gender: Male, Female) × 2 (Target Match: Target on babyface, Target on mature face) ANOVA on reaction time with 99.94% average accuracy (see **Table 1**).

**Table 1:** Means and standard deviations between the variables in Experiment 1.

Face	Target	Reaction	Accuracy
gender	match	time (ms)	(%)
Male	Babyface	359.77 ± 43.17	99.89 ± 0.00
	Mature face	357.68 ± 45.44	99.86 ± 0.00
Female	Babyface	360.58 ± 43.40	100.00 ± 0.00
	Mature face	365.45 ± 46.63	100.00 ± 0.00

*N = 44.*

There was a main effect on face gender (*F*_1,43_ = 6.13, *p* = 0.02, $\eta_{p}^{2}$ = 0.13), showing longer reaction time to target on the female faces than that on the male. Importantly, the interaction between face gender and target match was significant (*F*_1,43_ = 4.49, *p* = 0.04, $\eta_{p}^{2}$ = 0.10). By breaking up this interaction, a simple effect of target match was found for female faces (*F*_1,43_ = 4.14, *p* = 0.048, $\eta_{p}^{2}$ = 0.09), with faster responses to targets on babyfaces (361 ms) than mature faces (365 ms). In addition, the simple effect of Face Gender was observed when targets were shown on the mature face (*F*_1,43_ = 8.21, *p* < 0.01, $\eta_{p}^{2}$ = 0.16), showing worse performance with female faces (365 ms) than that with male faces (358 ms). Altogether, it seems that the female babyface and male mature face shortened the reaction time that contributed to this interaction.

### Discussion

In this experiment, we expected to detect if the babyface will attract the attention in a simple detection task. Without intervening participants’ visual selective attention, we analyzed their reaction time to targets presented on different face types and face genders. We found that it takes shorter to react when the target is on the male mature faces and female babyface. This result partially proved our assumption that the babyface has an influence on the reaction time in a simple detection task, but there are gender differences. For female faces, the babyface has an attention capture effect, which can attract the visual selective attention more rapidly. On the contrary, for male faces, it is the mature face that can be processed faster and catch visual selective attention.

In Experiment 1, there was no intervention on visual selective attention. Participants were free to observe with adequate attentional resource. We found that the babyface has an attention capture effect but with face gender bias. Since the attractiveness of face stimuli has been controlled during material selection, we consider that the differences of the reaction time indeed come from the influence of face types. Zebrowitz et al. (2009) believe that the babyface effect comes from baby schema (Lorenz, 1943) because of the attention capture effect of baby faces, but our results from the cognitive behavioral experiment showed that the theory of baby schema may not be the perfect explanation. The assumption that the adults’ babyface, similar to baby faces, will also attract visual selective attention seems to be only partly proved by the female babyface. Participants react more quickly to the target on the female babyface and male mature faces. One possible explanation is that during the procedure of Experiment 1, faces were demonstrated as a background. Participants catch sight of faces earlier before they notice the target letter. Since the faces were previewed for a randomized between 50 and 150 ms, the faces were processed in the earlier stage. In this earlier cognitive processing, female babyface and male mature faces may capture visual selective attention faster and gain more attentional resources, which results in shorter reaction time when the target appears on these faces. This finding confirmed that attention is necessary during the processing of face perception.

We need more evidence to prove that the babyface may have an influence on visual selective attention. In Experiment 1, faces are presented as a background, the target letter is overlapped on the face and no intervention on visual selective attention is conducted. If a face is presented outside the foveal vision, in other words, if the target letter and faces are separately displayed at the same time, can we still discover the influence of the babyface in a spatial cuing task with an intervention on visual selective attention? Thus, in the following experiment, we referred to an experimental paradigm created by Sui and Liu (2009) to conduct Experiment 2. To explore the competition of attentional resources, we presented the target and the face simultaneously in the brief time following a spatial cue. In this task, the cueing validity and inter-stimulus interval (ISI) were controlled to manipulate visual selective attention. Under a valid cue, the target letter is directly attended thus the distractive faces are unlikely to gain visual attention compared to an invalid cue. Also, since attentional resources are scarce for fast stimulus presentation, the ISI would have an substantial impact on attention resources allocated to the second stimuli (Vogel et al., 1998). Referring to Studies 1 and 3 of Sui and Liu (2009), we set ISI as 50 ms or 150 ms to influence the attentional resource allocation. Our hypothesis is that compared with mature faces, the babyface may spontaneously compete with an ongoing cognitive task for spatial attention and slows down the reaction time.

## Experiment 2

### Materials and Methods

#### Participants

Thirty-six undergraduate students from Tsinghua University, (14 females; ages 18–29 years; *M* ±*SD*, 21.86 ± 2.52 years) participated in the experiment. All participants had normal or corrected normal vision and were paid for their participation. At least 19 participants were needed and our sample size met the requirement. The study was approved by Tsinghua University Institutional Review Board and all participants gave informed consent.

#### Material and Stimuli

The material of study 1 was used in this experiment, however, the size of faces was edited into 400 pixels × 502 pixels.

**FIGURE 2:**
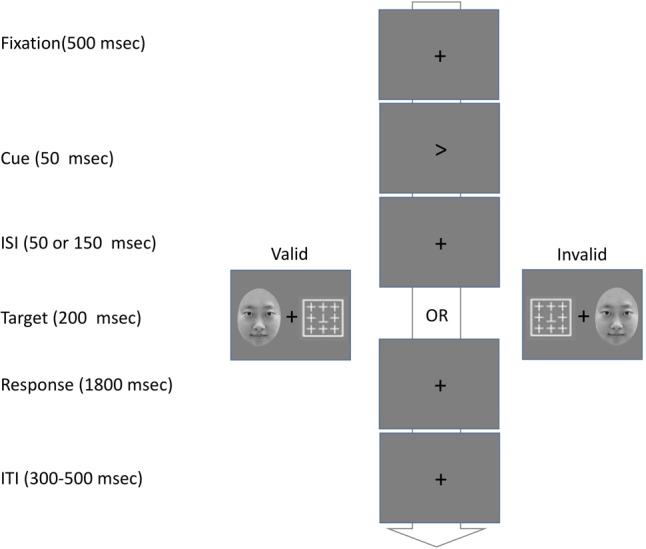
Illustration of the procedure used in the Experiment 2.

The stimuli were the same as that in experiment 1, except the 3.8° × 3.8° white boxes (see **Figure 2**). The outer edge of each box from the center display was 5.5° visual angle. A target letter “T” was surrounded by an array of eight distractor crosses (see **Figure 2**), which could be on one of the boxes. Each distractor or target was subtended at a 1.2° × 1.2° visual angle. The letter “T” was shown either upright or inverted.

#### Procedure

The procedure for each trial of this experiment is illustrated in **Figure 2**. Each trial began with a central fixation cross for 500 ms. It was then replaced by a 50-ms central cue, which pointed randomly to the right or the left box. In 80% of trials (valid trials), the cue indicated the target location. In the remaining 20% (invalid trials), the cue pointed to the opposite location of the target. The ISI between the cue and the target was 50 or 150 ms. A babyface or mature face was shown simultaneously with the target for 200 ms. The participants were asked to press ‘F’ for an upright target and ‘J’ for an inverted one in 2000 ms. The ITI was randomly set between 300 and 500 ms. Participants had to pass the practice trials with an accuracy over 80% before they took the 1280 experimental trials. There were 32 trials in each combined condition (2 Face genders × 2 Babyfacedness × 2 Cue validity × 2 ISI) in the experiment. Participants were allowed a short break after every 80 trials. It took about 60–70 min to finish the experiment.

### Results

#### Reaction Time

No participant was excluded according to the criterion as the same as that of Experiment 1. In the analysis of mean reaction times, trials with correct responses as well as reaction times in three standard deviations in each condition for individual participant were included. We conducted a 2 (Face gender: Male, Female) × 2 (Face Type: mature face, Babyface) × 2 (Cue Validity: Valid, Invalid) × 2 (ISI: 150 ms, 50 ms) Repeated Measures ANOVA on reaction time (see **Table 2**).

**Table 2:** Means and standard deviations between the variables in Experiment 2.

ISI (ms)	Cue validity	Face gender	Face type	Reaction time (ms)	Accuracy (%)
150	Invalid	Male	Babyface	601.45 ± 91.42	89.33 ± 9.92
			Mature face	616.61 ± 98.25	86.17 ± 14.64
		Female	Babyface	620.01 ± 99.13	86.33 ± 12.41
			Mature face	606.76 ± 93.56	86.08 ± 13.24
	Valid	Male	Babyface	522.50 ± 61.09	95.39 ± 2.69
			Mature face	521.53 ± 62.82	95.42 ± 3.03
		Female	Babyface	516.12 ± 63.29	96.22 ± 2.32
			Mature face	522.80 ± 61.38	95.06 ± 2.46
50	Invalid	Male	Babyface	624.26 ± 88.30	86.78 ± 12.63
			Mature face	622.13 ± 89.22	89.58 ± 11.18
		Female	Babyface	627.62 ± 90.45	87.03 ± 11.79
			Mature face	631.32 ± 85.59	84.89 ± 13.32
	Valid	Male	Babyface	552.92 ± 61.83	95.67 ± 3.46
			Mature face	553.67 ± 60.78	95.69 ± 2.82
		Female	Babyface	551.81 ± 61.96	95.72 ± 2.46
			Mature face	552.88 ± 60.97	95.83 ± 2.73

*N = 36.*

The analysis showed a main effect of ISI (*F*_1,35_ = 223.37, *p* < 0.01, $\eta_{p}^{2}$ = 0.87), with faster responses for a 150 ms cue-to-target ISI than a 50 ms ISI. The main effect of Validity was also observed (*F*_1,35_ = 54.09, *p* < 0.01, $\eta_{p}^{2}$ = 0.61), demonstrating slower reactions after an invalid cue than a valid cue. Notably, the four-way interaction was significant (*F*_1,35_ = 12.07, *p* < 0.01, $\eta_{p}^{2}$ = 0.26). Additionally, the three-way interaction ISI, Cue Validity, and Face Gender was significant (*F*_1,35_ = 6.21, *p* = 0.02, $\eta_{p}^{2}$ = 0.15), as was the interaction between Cue Validity, Face Gender, and Face Type (*F*_1,35_ = 6.15, *p* = 0.02, $\eta_{p}^{2}$ = 0.15). These interactions demonstrated the same pattern of results, with an influence of face type under flexible attentional resources, i.e., the invalid cueing condition and the long cueing time.

Specifically, in the four-way interaction, the simple main effect of face type was observed under long cueing and invalid cueing separately for males and females (see Supplementary Figure 1), indicating that visual selective attention modulates the effect of a babyface. We found that under long and invalid cueing, it takes longer to react on the target letter for male mature faces (*F*_1,35_ = 12.76, *p* < 0.01, $\eta_{p}^{2}$ = 0.27) and female babyfaces (*F*_1,35_ = 6.03, *p* = 0.02, $\eta_{p}^{2}$ = 0.15). Under long and valid cueing, it takes longer to react on female mature faces (*F*_1,35_ = 18.80, *p* < 0.01, $\eta_{p}^{2}$ = 0.35). Under short cueing time, there are no significant differences either with valid or invalid cueing.

#### Accuracy

In Experiment 2, accuracy is the other dependent variable in our analysis, since it can reflect the cognitive process of a much harder task. The criteria for data exclusion was the same as that of Experiment 1. We conducted a Repeated Measures ANOVA with 2 (Face gender: Male, Female) × 2 (Face Type: mature face, Babyface) × 2 (Cue Validity: Valid, Invalid) × 2 (ISI: 150 ms, 50 ms) design on accuracy (see **Table 2**).

There were main effects of Cue Validity (*F*_1,35_ = 22.14, *p* < 0.01, $\eta_{p}^{2}$ = 0.39) and Face gender (*F*_1,35_ = 5.00, *p* = 0.03, $\eta_{p}^{2}$ = 0.13), showing higher accuracy under valid than invalid conditions and better performance for presenting male faces than displaying female faces. Consistently, the four-way interaction was still observed (*F*_1,35_ = 9.55, *p* < 0.01, $\eta_{p}^{2}$ = 0.21), showing that the face type has an effect on visual selective attention and face gender bias appear (see Supplementary Figure 2). After analyzing the simple effect in the four-way interaction, we found that under long and valid cueing, the accuracy is lower for female mature faces (*F*_1,35_ = 7.06, *p* = 0.01, $\eta_{p}^{2}$ = 0.17). No significant differences are found under long and invalid cueing condition. Under short and invalid cueing time, the accuracy is higher for female babyface (*F*_1,35_ = 4.01, *p* = 0.05, $\eta_{p}^{2}$ = 0.10) and male mature faces (*F*_1,35_ = 9.36, *p* < 0.01, $\eta_{p}^{2}$ = 0.21). No significant differences are found under short and valid cueing condition.

### Discussion

In Experiment 2, by presenting the faces outside foveal vision, we manipulated visual selective attention by ISI and cue validity. We tested the influence of babyface on reaction time when the faces compete for spatial attention with an ongoing cognitive task. We found faster responses for a 150 ms cue-to-target ISI than a 50 ms ISI, shorter reaction time and higher accuracy for a valid cue than an invalid one. With more attention to the faces, i.e., the invalid cueing condition and the long cueing time, it takes longer to react on the target letter for the female babyface and male mature faces. This result partially agreed with the assumption and demonstrated that the babyface generates influences depending on the visual selective attention and face gender.

Obviously, we successfully intervened in the visual selective attention of participants by the cue. The influence of cue validity is significant, even if the cue is not completely related with the experiment task. Although during the debriefing, some participants reported that they tried to ignore the cue subjectively and purposely, the cue effect still influenced their reaction time and accuracy. With a valid cue, their reaction time can be significantly shortened and the accuracy can be enhanced. Otherwise, with an invalid cue, the reaction time is longer and the accuracy is lower.

It is easy to understand the influence of ISI on the reaction time. A short cueing time, ISI is 50 ms, implies insufficient attentional resource. Participants have less time to prepare and recognize the target, thus it takes longer for them to process, analyze and react in the later phase with sensory memory after the visual stimuli disappear. In this way, the reaction time under short cueing time condition is longer. In contrast, the long cueing time allows participants better preparation which ultimately shortens the reaction time.

Only under flexible attentional resources (the invalid cueing condition and the long cueing time) is the significant influence of face type and face gender prominently shown. In other words, only after the visual selective attention with awareness gets involved in the face processing, can the face type have an effect on our reaction time. It is also confirmed in Experiment 1 that attention is necessary during the processing of face perception.

The result of Experiment 2 is consistent with that of Experiment 1. In Experiment 1, we inferred that in the earlier cognitive processing, female babyface and male mature faces may capture visual selective attention faster and gain more attentional resource, which result in shorter reaction time when the target appears on these faces. Similar findings were also found in Experiment 2.

With long cueing time, ISI is 150 ms, and an invalid cue, the arrow indicating the face instead of the target, it takes longer to react on the target letter for female babyface and male mature faces. When the cue is invalid, the visual selective attention is first guided to face stimuli. While the face stimuli are irrelevant to the experimental task, participants need to distinguish the stimuli and shift their attention from the face to the target letter. In the process of discrimination, female babyface and male mature faces have an attention capture effect and it makes participants spend more time switching to the target letter, which leads to a longer reaction time. This can be considered as an attention disengagement effect of female babyface and male mature faces. Sui and Liu (2009) found that attractive faces also show an attention disengagement effect using a similar experimental paradigm. With an invalid cue, it also takes longer to switch from a more attractive face to the target. In our study, after controlling the attractiveness of face and instructing participants only focus on the central fixation, we found similar attention disengagement effect as Sui and Liu (2009), suggesting that the female babyface may be preferred by the participants, similar to the attractive faces (Dou et al., 2014; Zebrowitz et al., 2015; Zheng et al., 2016). The low accuracy of female mature faces under long and valid cueing were unexpected. As we discussed above, under this easy condition, the reaction time should be shortened and the accuracy should be enhanced. However, the results show an unexpected pattern: In this case, it is hard to deny a possible attention disengagement effect of female mature faces. Further studies are needed to explore deeply.

In previous lab behavioral experiments, Gorn et al. (2008) proposed that the babyface effect can be corrected by attention with awareness and deep processing, given long enough time. But, the results of Experiments 1 and 2 tell us that the correction of the babyface effect seems to be impossible in cognitive behavioral tasks requiring rapid response. In a limited short time, it is difficult to avoid the influence coming from facial configuration.

Now that the face type has an impact on visual selective attention with face gender bias on a supraliminal level, it is also worthwhile to study this influence without supraliminal access. Information can be attended to without being supraliminally perceived (Lamme, 2003; Koch and Tsuchiya, 2007), we assume that similar result will be found at the subliminal level in the Experiment 3. CFS (Tsuchiya and Koch, 2005; Jiang et al., 2006, 2007; Tsuchiya et al., 2006; Koch and Tsuchiya, 2007) was adopted, which is a powerful tool using high-contrast images continuously flashed at 10 Hz into one eye to suppress an image presented to the other eye. We expected that the babyface has an advantage in breaking suppression at the subliminal level.

## Experiment 3

### Materials and Methods

#### Participants

Forty-six undergraduate students from Tsinghua University, (23 females; ages 18–29 years; *M* ±*SD*, 22.50 ± 2.87 years) participated in the experiment. All participants had normal or corrected normal vision and were paid to attend. At least 43 participants were needed and our sample size met the requirement. The study was approved by Tsinghua University Institutional Review Board and all participants gave informed consent.

#### Stimuli

In this experiment, six faces (three female faces) were randomly selected from the high babyfaced group (H) and another six from the low babyfaced group (L) used in the experimental material of Experiments 1 and 2. The size of faces was edited into 4.1° × 6.2° on 22 inch LCD monitor (1280 × 1024, 100 HZ).

**FIGURE 3:**
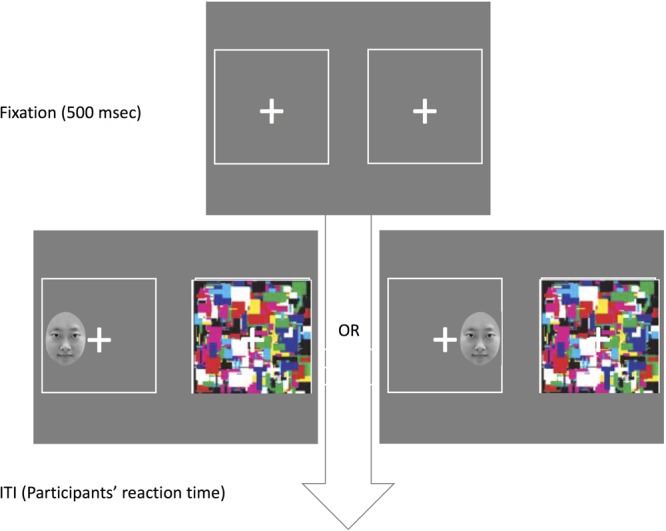
Illustration of the procedure used in the Experiment 3.

A central fixation cross (0.8° × 0.8°), two 10.7° × 10.7° white boxes, distinct images flashed successively at 10 Hz into one eye (4.1° × 6.2°) were presented (see **Figure 3**). Matlab and the Psychophysics Toolbox (Brainard and Vision, 1997; Pelli, 1997) was used to control the flow of the experiment and to collect response data. Participants were tested individually in a quiet and closed room in a dim light.

#### Procedure

The participant’s viewing position was set 57 cm away from the computer monitor with an adjustable chin rest. The images presented to the two eyes were displayed side by side on the monitor and fused using mirror stereoscopes mounted on the chin rest. A central cross was always presented to each eye, serving as the fixation point. First, participants were asked to watch the left side of the monitor by left eye and the right side by right eye. Two 10.7° × 10.7° white boxes with a same image were displayed on both sides. The researcher adjusted the mirror stereoscopes until the images from participants’ two eyes were overlapped perfectly.

The procedure for each trial of the experiments is illustrated in **Figure 3**. Each trial began with a central fixation cross. Then, a distinct image flashed successively at 10 Hz was presented into one eye and a face into the other eye, randomly on left or right eye. At the beginning of each trial, participants can only recognize a flash image. After a while, the whole face or some part of the face gradually will come into the participants’ mind. The face will be presented randomly on the left or right side of the central fixation cross. Participants were asked to react to the position of the face. If it is on the left side, they press the left arrow key; if it is on the right side, they press the right arrow key.

Participants had to pass 20 practice trials with accuracy over 90% before they took the 720 experimental trials. Each of the four conditions (2 Face genders × 2 Babyfacedness) in the experimental trials had 60 trials and every three faces of each condition was shown to all the participants. They were given short breaks after every 60 trials. It took about 90 min to finish the experiment.

### Results

To reject data outliers, we excluded trials in which the reaction time was longer than 10 s (this value was more than three standard deviations away from the sample mean). No participant was excluded. We conducted a 2 (Face gender: Male, Female) × 2 (Face type: Babyface, Mature face) Repeated Measures ANOVA on reaction time with 95.65% average accuracy.

**FIGURE 4:**
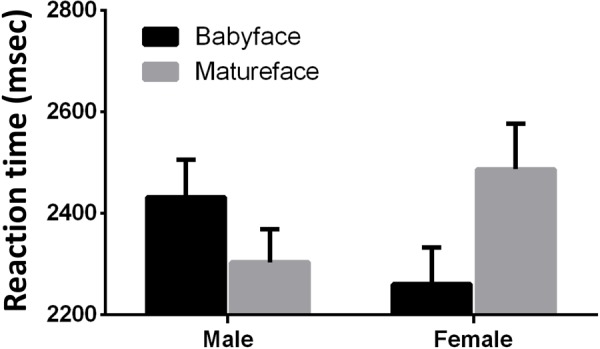
Interaction effects of the reaction time in the Experiment 3. Reaction time (msec, *M* ±*SD*) on male babyface is 2432.14 ± 500.86, with accuracy (%, *M* ± *SD*) 95.74 ± 0.07; reaction time on male mature face is 2303.84 ± 441.41, with accuracy (%) 95.33 ± 0.87; reaction time on female babyface is 2260.95 ± 491.56, with accuracy (%) 95.98 ± 0.08; reaction time on female mature face is 2487.12 ± 606.25, with accuracy (%) 95.53 ± 0.80.

The variables of babyfacedness had a main effect on the reaction time. It takes shorter to react to a babyface (*F*_1,45_ = 4.30, *p* = 0.04, $\eta_{p}^{2}$ = 0.09). The interaction effect between face gender and babyfacedness is significant (*F*_1,45_ = 35.88, *p* < 0.01, $\eta_{p}^{2}$ = 0.44). After analyzing the simple effect, we found that it takes participants less time to react on both the male mature face (*F*_1,45_ = 16.00, *p* < 0.01, $\eta_{p}^{2}$ = 0.26) and the female babyface (*F*_1,45_ = 27.86, *p* < 0.01, $\eta_{p}^{2}$ = 0.38) (see **Figure 4**).

### Discussion

In Experiment 3, we aimed to research the influence of the babyface on visual selective attention without supraliminal access. The time of the whole face or some part of the face breaking the CFS represents how fast the face is processed at the subliminal level. The assumption that the babyface has an advantage in CFS task is partially proved. It is the female babyface and male mature face that break the suppression faster.

This result is consistent with the results of Experiments 1 and 2. The babyfacedness of faces has a significant influence on the reaction time, but the effect varies with face genders. The female babyface and male mature face can break the suppression more quickly and come into participants’ mind earlier, which leads to the shorter reaction time.

At a subliminal level, with adequate attention resource, the female babyface still has an effect on the reaction time. Facial configuration has a deep influence beyond our imagination on our behavior, both supra- and sub- liminally. In this way, we need more effort and long enough time to correct the babyface effect. Otherwise, we will be affected by the face subliminally in the earlier stage.

## General Discussion

We researched the attention processing mechanism of the babyface both supra- and sub- liminally. Our findings revealed that the babyface affects our cognitive behavior, depending on visual selective attention but with face genders bias. It is the female babyface and male mature face that have an attention capture effect and attention disengagement effect at a supraliminal level, and also an advantage breaking the suppression at a subliminal level. Taken together, the female babyface and male mature face can glue and capture more visual selective attention and make it difficult to take your eyes off of it.

The results of these three experiments are reliable and internally consistent. In Experiment 1, we inferred that the female babyface and male mature face can capture more attention earlier. Similar findings were shown in Experiment 2 but limited under invalid and longer ISI conditions. Cue validity and ISI are related to attention orientation and sufficiency. Participants’ attention was led to irrelevant face stimuli by an invalid cue. With longer ISI, they have more time to prepare. After manipulating the visual selective attention in Experiment 2, stronger evidence is presented that a female babyface and male mature face presented outside foveal vision can catch participant’s eyes and compete with an ongoing cognitive task in a spatial cuing task. Furthermore, coherent results were investigated with the CFS experiment. We found that the female babyface and male mature face have an advantage breaking suppression. A possible explanation is that the female babyface and male mature face glued our visual selective attention even without consciousness. Both in Experiments 1 and 3, flexible attentional resources were applied without intervention. Either with or without awareness, it is always the female babyface and male mature face that show the significant influence on participants’ response. From the above, these three experiments illustrate that the babyface influences our behavior by affecting the visual selective attention supra- and sub-liminally, with a facial gender bias.

Our findings partly verified previous research. The preference for babyface is also found in our study, which is consistent with previous research (Dou et al., 2014; Zebrowitz et al., 2015; Zheng et al., 2016). No influence of participants’ gender on the perception of babyface was found previously^4^, but gender difference of the stimuli faces does bias the influence of a babyface on our behavior. We cannot entirely prove the hypothesis of Zebrowitz et al. (2009) that the attraction and retention of visual selective attention is the reason of a babyface generating the babyface effect. Because we only find some proof for the female babyface. This inspires us to take the evolutionary tendency into account to explain the babyface effect and understand facial perception, instead of only considering the theory of baby schema. Because of similar facial features with babies, a babyface makes a younger impression, and the young are mostly related to stronger fertility. In mate selection, the male is concerned more about the ability to have offspring, and the female concerned more about obtaining supportive resources (Buss and Barnes, 1986; Buss, 1989). A lady with a babyface looks younger, cute, charming, innocent and kind (Berry and McArthur, 1985; McArthur and Berry, 1987; Zebrowitz et al., 1992, 1993, 2015; Albright et al., 1997; Zebrowitz and Montepare, 2005; Zebrowitz, 2006; Luevano and Zebrowitz, 2007) and more gorgeous. A man with a mature face, such as a sharp chin and dark eyebrows, is attractive to women. Because these characters may imply physical health, reproductive ability, resources occupation, higher social status, leadership and power. They may offer stronger protection (Keating, 1985), therefore, these potential ruling elite and leaders will be discovered soon. This rule may be widely accepted, which may possibly be the reason why there is no participants’ gender difference.

## Conclusion

The cognitive behavior experiments are more objective and convincing with direct behavioral evidence instead of self-report. Furthermore, our findings are stable, consistent, and verified by different experiment paradigms. We explored the attention processing mechanism of the babyface and confirmed its qualifications. However, we should also consider cultural differences in the definition of the babyface and in inferences regarding the babyface in different cultural contexts. More cross-cultural studies should be conducted to research whether it is universal or it is special in the East Asian culture which advocates the obedience of the female. Additionally, the limitation of face stimuli may still exist, such as age controlling, requiring further studies. Our study researched the face perception at the earlier stage of attention processing. Future studies may focus on the attention processing by eye tracking and ERP technology. These studies may offer more objective physical proof in confirming or disconfirming our explanation of the female babyface and male mature face as gluing visual selective attention.

## Author Contributions

WZ developed the study concept with KP and the experimental paradigm with TL and C-PH. WZ, TL, and C-PH conducted the experiments and collected the data. WZ and TL performed the data analysis and interpretation under the supervision of KP and drafted the manuscript. TL, C-PH, and KP provided critical revisions. All authors contributed to discussion of the manuscript and approved the work for publication.

## Conflict of Interest Statement

The authors declare that the research was conducted in the absence of any commercial or financial relationships that could be construed as a potential conflict of interest.

## Footnotes

1 www.babyface-book.net

2 First, the website (www.babyface-book.net) was referred to filter 540 faces (240 female, age: 22–45 years old), half of which were highly babyfaced. 31 undergraduate students (20 female; ages 18–23 years; *M* ±*SD*, 20.10 ± 2.02 years) from Tsinghua University evaluated the Attractiveness and Babyfacedness of these faces on a 7-point scale. Each face was rated at least four times by different participants. The babyfacedness of these faces from human evaluation and website measurement were positively correlated, *r*(317) = 0.36, *p* < 0.01. According to previous studies, attractiveness is positively related with babyfacedness. In order to control this confounding variable, faces with similar attractiveness but a big difference in babyfacedness between high babyfaced group (H) and low babyfaced group (L) were chosen. 147 female faces were selected with 72 babyfaces in the H group and 75 mature faces in the L group. 170 male faces were selected with 82 babyfaces in the H group and 88 mature faces in the L group. When we look back, the perceived age of these 317 chosen faces were rated by the program of #HowOldRobot (cn.how-old.net) developed by Microsoft, there is no significant difference among four groups (female babyface, female mature face, male babyface and male mature face), *F*(3,313) = 0.33, *p* = 0.80, $\eta_{p}^{2}$ = 0.00.

3 We analyzed Z score of the attractiveness of faces (female babyface, female mature face, male babyface, and male mature face) by human evaluation and found no significant difference, *F*(3,313) = 0.14, *p* = 0.94, $\eta_{p}^{2}$ = 0.00. *Z* score of a combined score [Combined score = babyfacedness by website measurement/(8 – babyfacedness by human evaluation)] of the babyfacedness was used to analyze. Apparently, significant differences were found, *F*(3,313) = 40.77, *p* < 0.01, $\eta_{p}^{2}$ = 0.28. With *post hoc* tests (Tamhane adjusted), we found that at the 0.01 significance level, the babyfacedness is significant difference between female babyface (*M* = 0.79, *SD* = 1.17) and mature face (*M* = -0.43, *SD* = 0.72), *SE* = 0.16, *p* < 0.01, 99% *CI* [0.70, 1.74]; also, male babyface (*M* = 0.27, *SD* = 0.94) and mature face (*M* = -0.53, *SD* = 0.48), *SE* = 0.12, *p* < 0.01, 99% *CI* [0.42, 1.17]. No significant difference was found between female babyface (*M* = 0.79, *SD* = 1.17) and male babyface (*M* = 0.27, *SD* = 0.94), *SE* = 0.17, *p* = 0.02, 99% *CI* [-0.03, 1.07]; also, female mature face (*M* = -0.43, *SD* = 0.72) and male mature face (*M* = -0.53, *SD* = 0.48), *SE* = 0.10, *p* = 0.91, 99% *CI* [-0.41, 0.22].

4 We considered participants’ gender as a variable in the further analysis of the three experiments in our study, but no significant effect from participants’ gender was found. Thus, the variable of participants’ gender wasn’t included in the results reported.
