# Supplementary material for: Glued to Which Face? Attentional Priority Effect of Female Babyface and Male Mature Face
Source: Front Psychol. 2018 Mar 6;9:286. doi: 10.3389/fpsyg.2018.00286 (PMC5845684; doi:10.3389/fpsyg.2018.00286)
Supplement: Supplementary file 1 [file Presentation_1.PDF]

Supplementary Material

Glued to Which Face?

Attentional Priority Effect of Female Babyface and Male Mature Face

Wenwen Zheng[1], Ting Luo[1], Chuan-Peng Hu[1], Kaiping Peng[1*]

[1]Department of Psychology, Tsinghua University, Beijing 100084, China.

*** Correspondence:**
Corresponding Author: pengkp@mail.tsinghua.edu.cn

1 Supplementary Figures

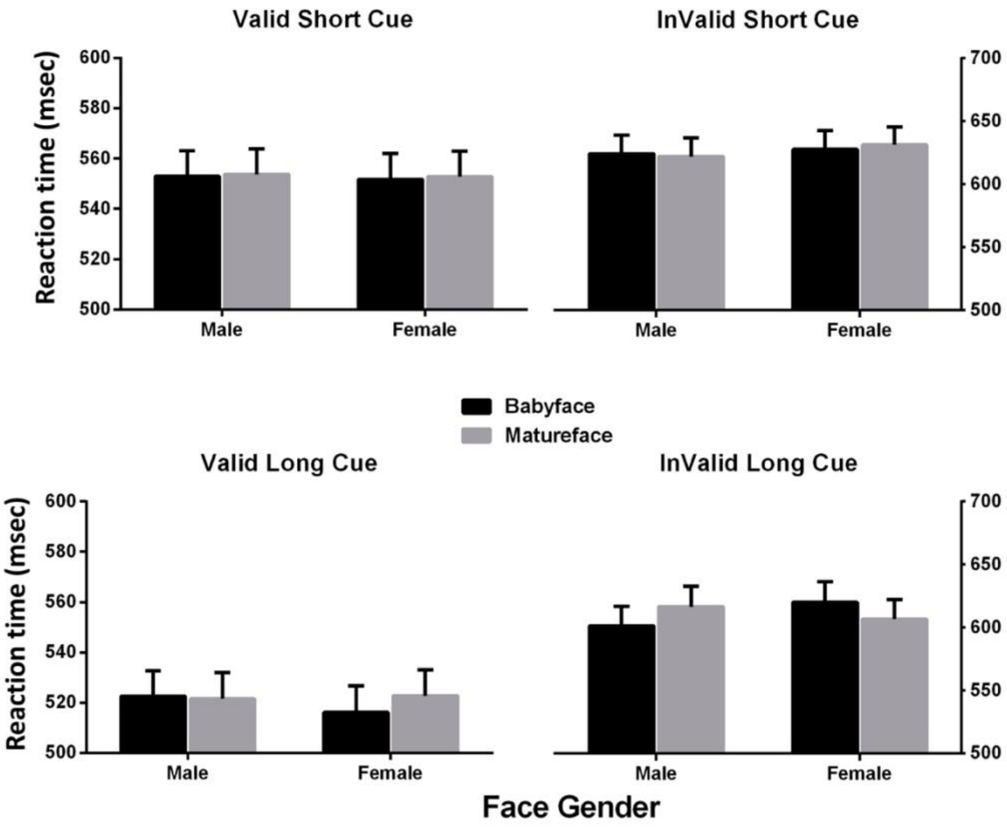

Supplementary Figure 1. Four-way Interaction Effects of the Reaction Time ($M \pm SD$) in the Experiment 2.

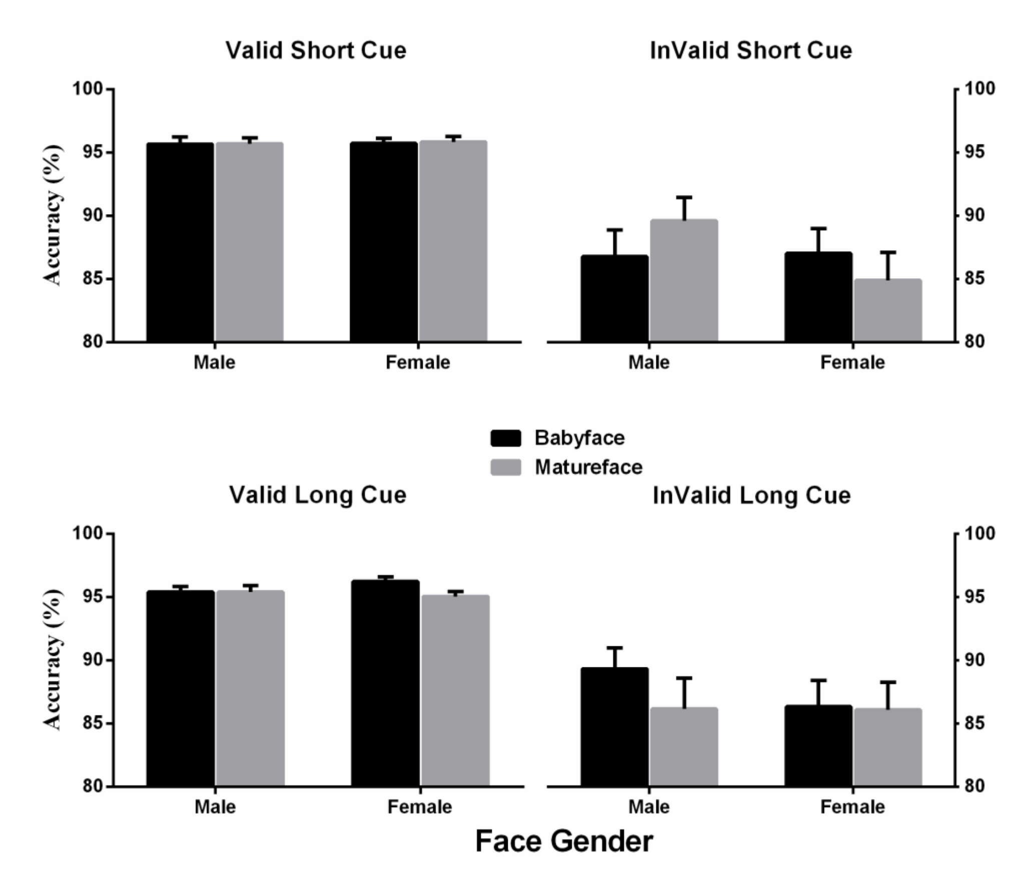

Supplementary Figure 2. Four-way Interaction Effects of the Accuracy ($M \pm SD$) in the Experiment 2.
